# Integrative analysis of competitive endogenous RNA network reveals the regulatory role of non-coding RNAs in high-glucose-induced human retinal endothelial cells

**DOI:** 10.7717/peerj.9452

**Published:** 2020-06-29

**Authors:** Nan-Jue Cao, He-Nan Liu, Feng Dong, Wei Wang, Wei Sun, Gang Wang

**Affiliations:** 1 Department of Ophthalmology, The Fourth Affiliated Hospital, Zhejiang University School of Medicine, Yiwu, Zhejiang, Peoples R China; 2 Department of Ophthalmology, Shengjing Hospital, China Medical University, Shenyang, Liaoning, Peoples R China; 3 Department of Ophthalmology, The First Affiliated Hospital, Zhejiang University School of Medicine, Hangzhou, Zhejiang, Peoples R China; 4 School and Hospital of Stomatology, China Medical University, Liaoning Provincial Key Laboratory of Oral Diseases, Shenyang, Liaoning, Peoples R China; 5 Department of Radiology, The Fourth Affiliated Hospital, Zhejiang University School of Medicine, Yiwu, Zhejiang, Peoples R China; 6 Department of Rheumatology, The Fourth Affiliated Hospital, Zhejiang University School of Medicine, Yiwu, Zhejiang, Peoples R China

**Keywords:** Bioinformatics, Human retinal endothelial cells, Diabetic retinopathy, Microarray, Competitive endogenous RNA network, Long non‑coding RNAs, miRNAs

## Abstract

**Background:**

Increasing evidence has suggested that non-coding RNAs (ncRNAs) play critical roles in the pathogenesis of diabetic retinopathy (DR), but their underlying mechanisms remain unclear. The purpose of this study was to determine latent key genes and to structure a competing endogenous RNA (ceRNA) regulatory network to discover the potential molecular mechanisms governing the effects of high glucose on human retinal endothelial cells (HRECs).

**Methods:**

We obtained microarray data for long non-coding RNA (lncRNA) and mRNA of high-glucose-induced HREC samples from NCBI GEO datasets. The ceRNA network was screened using intersecting prediction results from miRcode, TargetScan, miRTarBase and miRDB. The protein–protein interaction (PPI) network was constructed using the Search Tool for the Retrieval of Interacting Genes and hub genes were obtained using the cytoHubba app. The ClusterProfiler package was applied for performing Gene Ontology (GO) and Kyoto Encyclopedia of Genes and Genomes (KEGG) pathway analysis. The expression of key RNAs was verified using the qRT-PCR method. A key ceRNA subnetwork was constructed based on the criticality of the genes and its binding sites were verified by luciferase reporter assay. The viability and apoptosis of HRECs were tested using the transfection of the miR-449c inhibitor.

**Results:**

A total of 3,328 lncRNAs and 2,017 mRNAs were screened for differentially expressed (DE) profiles. The newly constructed ceRNA network was composed of 410 lncRNAs, 35 miRNAs and 122 mRNAs. The 10 hub genes were identified through the PPI network. GO and KEGG analysis revealed that DE mRNAs were mainly related to the positive regulation of the mRNA catabolic process, cell polarity, and the G1/S transition of mitotic and cell cycle signaling pathways. QRT-PCR was used to verify RNAs and the most important genes were screened out. A key ceRNA subnetwork OIP5-AS1/miR-449c/MYC was established. The binding site was verified by luciferase reporter assay. The expression levels of OIP5-AS1 and MYC increased after miR-449c inhibitor transfection, miR-449c decreased, HRECs activity increased, and apoptosis decreased, compared with the control group.

**Conclusion:**

We successfully built the key ceRNA subnetwork, OIP5-AS1/miR-449c/MYC, by applying the GEO database for data analysis and mining. The results from the ceRNA network allow us to better understand the effect of ncRNAs on HRECs under hyperglycemic conditions and the pathogenesis of DR.

## Introduction

Diabetic retinopathy (DR) is one of the most common complications of diabetes mellitus (DM) and is a leading cause of vision impairment and blindness worldwide (Cheung, Mitchell & Wong, 2010). DR is reaching epidemic levels globally due to the increasing prevalence of DM (Scanlon, Aldington & Stratton, 2013). The early stages of DR are characterized by pericyte necrosis and retinal endothelial cell (REC) dysfunction. As the disease progresses, vascular leakage, a clinical characteristic of DR, becomes apparent and may result in diabetic macular edema, the most common cause of vision loss in DR (Roy et al., 2017). The mechanism of abnormal metabolism of various cells due to hyperglycemia is not clear. It is recognized that REC damage is highly related to the incidence of DR (Xie et al., 2008; Mohammad et al., 2019), so understanding the effect of high glucose on RECs is significant to determining the incidence of DR and to discovering effective targeted therapies, including latent key genes and DR-resistant targets.

MicroRNAs (miRNAs) are noncoding transcripts of approximately 22 nucleotides that are important in the post-transcriptional regulation of protein coding genes by direct translational repression, mRNA destabilization and/or mRNA cleavage (Schirle, Sheu-Gruttadauria & MacRae, 2014). Long non‑coding RNAs (lncRNAs) are noncoding transcripts that are typically longer than 200 nucleotides and that perform multiple functions. lncRNAs may act as competitive endogenous RNA (ceRNA) by miRNA sponges, decreasing miRNAs regulatory influence upon mRNAs (Bartel, 2009; Mercer, Dinger & Mattick, 2009). Growing evidence has indicated that non-coding RNAs (ncRNAs), especially miRNAs and lncRNAs, are unconventionally expressed in DR and may play an important role in the occurrence and development of DR through the regulation of gene expression at the epigenetic, transcriptional, or post-transcriptional levels (Gong & Su, 2017; Biswas, Sarabusky & Chakrabarti, 2019). However, the latent mechanism of the ceRNA regulatory network in HG-induced human retinal endothelial cells (HRECs) is yet to be determined. The determination of pivotal ceRNA factors that forecast HG HRECs may lead to the more accurate early diagnosis, prevention, or development of targeted therapeutic treatments.

We analyzed a microarray dataset (GSE122189) of lncRNAs and mRNAs and compared HG-induced HRECs to those cultured at normal-glucose levels. We developed a whole triple network according to the ceRNA theory by applying data from the GEO website, as lncRNA and mRNA form a relation by overlapping the identical miRNA, and also revealed essential genes. Our results indicated the lncRNAs–miRNAs–mRNAs network offers novel hypothesis mechanisms and latent treatment targets for the effect of hyperglycemia on HRECs.

## Materials and Methods

### Microarray data

Microarray data from the GSE122189 dataset was downloaded from the GEO (https://www.ncbi.nlm.nih.gov/geo/) database. The GSE122189 dataset includes 2 normal glucose (NG) samples (HRECs cultured in 5 mmol/l glucose conditions for 48 h) and 2 HG group samples (HRECs cultured in 25 mmol/l glucose conditions for 48 h) (Thomas et al., 2019). The GPL16956 platform was hybridized for lncRNA–mRNA microarray analysis using an Agilent-045997 Arraystar human lncRNA microarray V3. Approximately 28,035 lncRNAs and 21,407 mRNAs were detected using this microarray.

### Differential expression analysis

We applied R software limma and sva software packages for the analysis of microarray data to correct the microarray background and normalize the initial data (Leek et al., 2012; Ritchie et al., 2015). We then replaced the probe name in the microarray with the gene symbol, removed the probe without the gene symbol and calculated the average value of gene symbols with multiple probes. The differently expressed (DE) lncRNAs and mRNAs were screened using limma package in R software with an adjusted *P*-value < 0.05 and the criterion of |log2 (fold change)| > 1. Hierarchical cluster analysis was conducted using the pheatmap package in R software. The clustering was performed using the Euclidean distance method (Draisma et al., 2016).

### Construction of the ceRNA regulatory network

miRcode was applied to forecast lncRNA–miRNA interactions (Jeggari, Marks & Larsson, 2012) and TargetScan, miRTarBase and miRDB were used to predict miRNA–mRNA interactions (Agarwal et al., 2015; Wong & Wang, 2015; Chou et al., 2018) to discover whether lncRNA–miRNA–mRNA existed as a competing endogenous regulating network. Only the mRNAs identified by the above three websites were designated as candidate mRNAs and were intersected with DE mRNAs to screen the mRNAs targeted by miRNAs. The DE lncRNAs and mRNAs were used for ceRNA construction. Visualization of the ceRNA network was constructed using Cytoscape v3.7.2 (Shannon et al., 2003).

### Construction of the protein–protein interaction network and identification of hub genes

The Search Tool for the Retrieval of Interacting Genes (STRING) was used to discover the protein–protein interaction (PPI) network among mRNAs that were in the ceRNA regulatory network, which can provide comprehensive interactions among proteins and genes (Szklarczyk et al., 2019). A combined score of ≥ 0.4 was chosen for PPI network construction and disconnected nodes were hidden in the network for further visualization. The PPI network was visualized by applying Cytoscape v3.7.2 (Shannon et al., 2003). The cytoHubba app was used to excavate the hub genes of the obtained PPI network (Chin et al., 2014).

### Functional and pathway enrichment analysis

To assess the function of genes in the ceRNA network, Gene Ontology (GO) analyses and Kyoto Encyclopedia of Genes and Genomes (KEGG) pathway enrichment analyses were annotated using the clusterProfiler package of R software (Kanehisa & Goto, 2000; Yu et al., 2012; Gene Ontology Consortium, 2015). An adjusted *P*-value < 0.05 was set as the cutoff criterion.

### Cell culture

HRECs (Olaf Pharmaceuticals, Worcester, MA, USA; https://www.interchim.fr/ft/E/EOC230.pdf) were cultured in an endothelial cell growth medium (EGM)-2 (Lonza, Walkensville, MD, USA) with 10% fetal bovine serum (HyClone, Logan, UT, USA). Human embryonic kidney (HEK)-293T cells (Stem Cell Bank, Chinese Academy of Sciences, Shanghai, China) were cultured in DMEM (HyClone, Logan, UT, USA) with 10% fetal bovine serum (HyClone, Logan, UT, USA). The culture medium was replaced every 1–2 days. The cells were passaged at a ratio of 1:3 once they grew to 85–90% confluence. After 24 h of culturing in serum-free EGM-2, HRECs were cultured in different glucose concentrations (NG-treated was in 5 mmol/l glucose and HG-induced was in 25 mmol/l glucose) for 48 h.

### Cell transfection

HRECs were transfected with the has-miR-449c-5p (miR-449c) inhibitor or its negative control (NC) (GenePharma, Shanghai, China) applying the Lipofectamine^TM^ 2000 Transfection Reagent (Invitrogen, Carlsbad, CA, USA) according to the manufacturer’s instructions. After transfection, HRECs were cultured with 25 mmol/L glucose for 48 h and then collected for further analysis.

### Quantitative real-time PCR (qRT-PCR)

Total RNA was isolated using RNAiso Plus (Takara, Shiga, Japan). cDNA was generated by reverse transcription from total RNA using the PrimeScript™ RT Reagent Kit (Takara, Shiga, Japan). Real-time PCR was performed using TB Green® Premix Ex Taq™ II (Takara, Shiga, Japan). qRT-PCR primer sequences are presented in Table S1. The relative expression level was analyzed using the 2^−ΔΔCt^ method. GAPDH mRNA and U6 snRNA were chosen as internal controls.

### Luciferase reporter assay

The wild-type (WT) and mutant (MUT) OIP5-AS1 and MYC binding sites of miR-449c were synthesized into pmirGLO vectors (Promega, Madison, WI, USA) to construct the recombinant reporters pmirGLO-OIP5-AS1-WT, pmirGLO-OIP5-AS1-MUT, pmirGLO-MYC-WT, and pmirGLO-MYC-MUT, respectively. The reporters were then co-transfected together with miR-449c mimic or mimic NC to HEK-293T cells. Luciferase activity was analyzed using the Dual-Luciferase Reporter Assay System according to the manufacturer’s instructions (Promega, Madison, WI, USA) after 48 hours’ transfection. Firefly luciferase activity was normalized to that of Renilla luciferase.

### Reconstruction of the key ceRNA subnetwork

Every triple correspondence in the above ceRNA was refiltered to establish a novel subnetwork. The genes with the greatest expression differences were screened out according to the top five most significant DE lncRNAs and verification of the hub genes. We made corresponding miRNA predictions and their binding sites were verified. The key ceRNA subnetwork was reconstructed.

### Cell viability

Cells were cultured for 6, 12, 24 and 48 h after transfection and cell viability was measured using Cell Counting Kit-8 (CCK-8) according to the manufacturer’s instructions (Solarbio, Beijing, China). The Sunrise microplate reader (Tecan Group, Männedorf, Switzerland) measured the absorbance at 450 nm.

### Cell apoptosis

Cells were cultured for 48 h after transfection and then cell apoptosis was measured using Annexin V-FITC Apoptosis Detection Kit (BD Biosciences, San Diego, CA, USA) according to the manufacturer’s instructions for the FACS Vantage Flow cytometer (BD Biosciences, San Diego, CA, USA). The data were analyzed to determine the ratio of cell apoptosis using CellQuest software (BD Biosciences, San Diego, CA, USA).

### Statistical analysis

Data were calculated using Independent Samples *t*-test. All qRT-PCR values were represented as the mean ± standard deviation (SD). Differences with *P* < 0.05 were considered statistically significant.

## Results

### Differentially expressed RNAs

**Figure 1:**
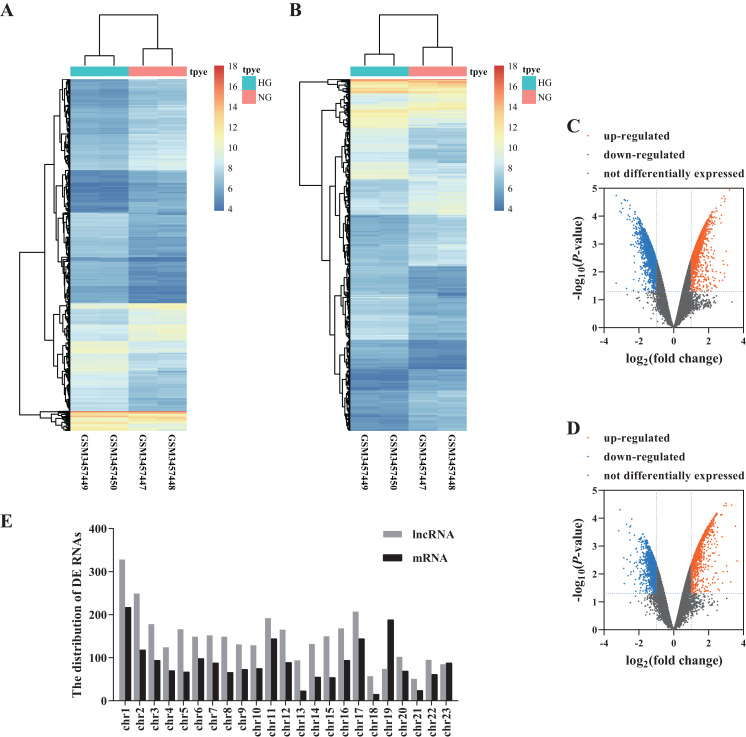
Differential profiling of lncRNAs and mRNAs in the HG and NG-induced HRECs from microarray data. The cluster heat map of DE lncRNAs (A) and mRNAs. (B) Volcano plots presented identified DE lncRNAs (C) and mRNAs (D) with a fold change (FC) ≥ 1.0 and an FDR < 0.05. Orange represents up-regulated genes, blue represents down-regulated genes. The distribution of DE RNAs on the human chromosomes (E).

We analyzed the microarray data and obtained 3,328 DE lncRNAs (1,645 up-regulated and 1,683 down-regulated) and 2,017 DE mRNAs (1,027 up-regulated and 990 down-regulated) (Figs. 1A–1D; Data S1). The distribution of DE RNAs on the human chromosomes is shown in Fig. 1E.

### ceRNA regulatory network construction

**Figure 2:**
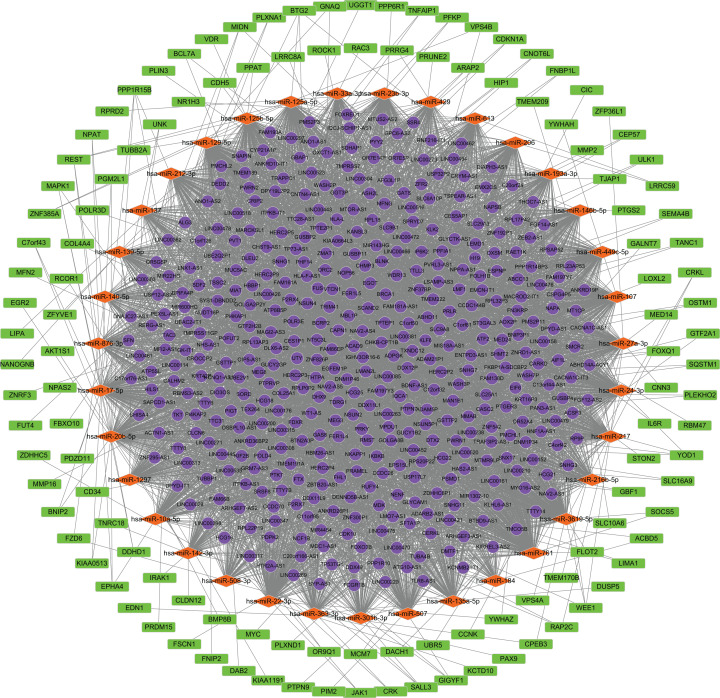
Construction of the ceRNA network. Round, diamond and rectangle represent lncRNAs, miRNAs and mRNAs, respectively.

We predicted the miRNAs targeted by DE lncRNAs and DE mRNAs. Using miRcode, we found that 207 miRNAs were targets of 401 DE lncRNAs. TargetScan, miRTarBase and miRDB, were applied to the 207 miRNAs to predict the targeted mRNAs. As a result, 1,296 mRNAs were found to correspond with 207 miRNAs. The 1,296 mRNAs intersected with 2,017 DE mRNAs. Finally, 122 mRNAs were confirmed. 410 lncRNAs, 35 miRNAs and 122 mRNAs were screened for building the ceRNA network. (Fig. 2; Data S2).

### PPI network construction and hub genes analysis

**Figure 3:**
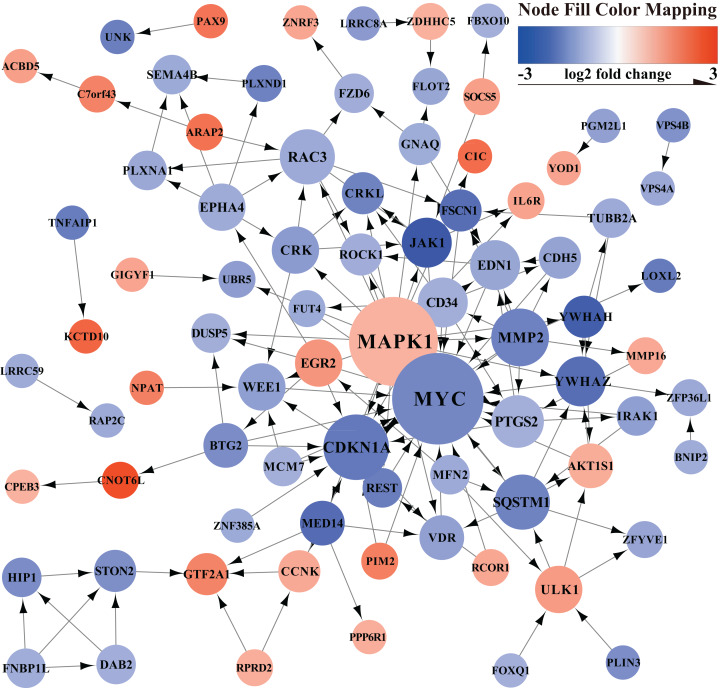
PPI network. Orange represents up-regulated genes, blue represents down-regulated genes.

The PPI network of mRNAs in the ceRNA regulatory network is shown in Fig. 3. The PPI network showed the relationship between proteins and the significant genes (MYC, MAPK1, CDKN1A, MMP2, SQSTM1, RAC3, PTGS2, CD34, YWHAZ, JAK1) were determined using Cytohubba in Cytoscape (Table 1).

**Table 1:** PPI network of the top 10 hub genes.

Rank	Name	Score	GeneID	Official Full Name
1	MYC	23	4609	MYC proto-oncogene, bHLH transcription factor
2	MAPK1	22	5594	mitogen-activated protein kinase 1
3	CDKN1A	13	1026	cyclin dependent kinase inhibitor 1A
4	MMP2	10	4313	matrix metallopeptidase 2
5	SQSTM1	9	8878	sequestosome 1
5	RAC3	9	5881	Rac family small GTPase 3
7	PTGS2	8	5743	prostaglandin-endoperoxide synthase 2
8	CD34	7	947	CD34 molecule
8	YWHAZ	7	7534	tyrosine 3-monooxygenase/tryptophan 5-monooxygenase activation protein zeta
8	JAK1	7	3716	Janus kinase 1

### Functional and pathway enrichment analysis

**Figure 4:**
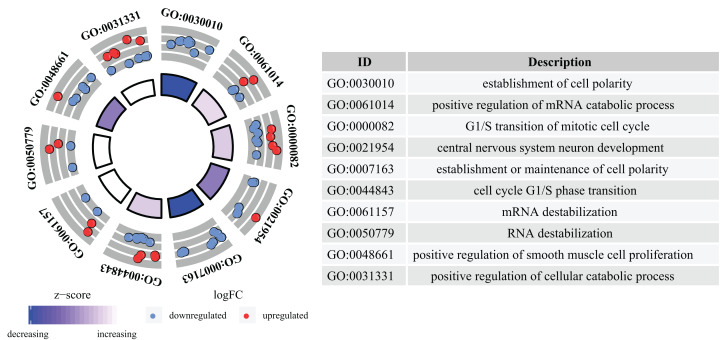
GO analysis of aberrantly expressed genes. GO annotations of mRNAs with the top 10 enrichment scores. Different colored trapezoids represent different *Z*-scores, red circles represent up-regulated genes, and blue circles represent down-regulated genes.

GO analysis determined that a total of 109 GO terms were significantly enriched. The top 10 enriched GO terms were: the establishment of cell polarity, positive regulation of mRNA catabolic process, G1/S transition of mitotic cell cycle, central nervous system neuron development, establishment or maintenance of cell polarity, cell cycle G1/S phase transition, mRNA destabilization, RNA destabilization, positive regulation of smooth muscle cell proliferation, and positive regulation of cellular catabolic process. The top 10 GO analysis results are presented in Fig. 4.

**Figure 5:**
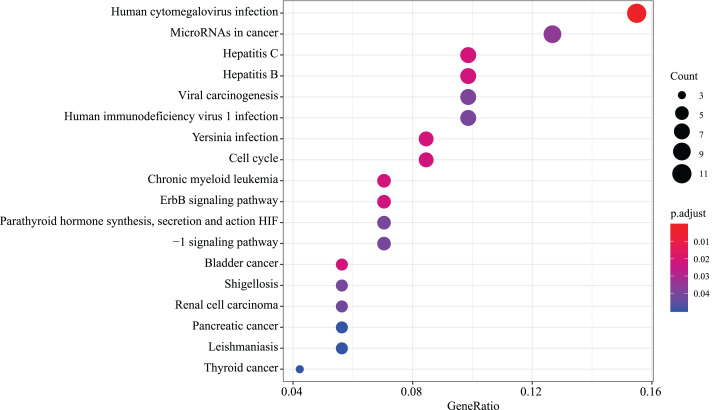
KEGG pathway analysis of aberrantly expressed genes. 18 KEGG pathway enrichment analysis were significantly enriched. Different circle sizes represent different gene numbers, and different colors stand for different adj. *P*-value. The abscissa indicates different GeneRatio.

We found that 18 KEGG pathway terms were significantly enriched. The top 10 pathways with the most meaningful enrichment were: human cytomegalovirus infection, hepatitis C, bladder cancer, chronic myeloid leukemia, hepatitis B, yersinia infection, cell cycle, erbB signaling pathway, microRNAs in cancer and viral carcinogenesis. KEGG enrichment analysis pathways are presented in Fig. 5 and Data S3.

### Candidate RNAs validation and key ceRNA subnetwork confirmation

**Figure 6:**
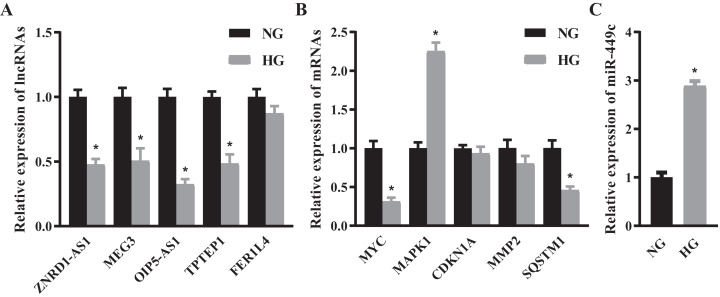
QRT-PCR results of selected RNAs. QRT-PCR for the top 5 DE lncRNAs (A), hub genes (B) and miR-449c (C) of HRECs in the NG and HG groups. *P < 0.05.

**Figure 7:**
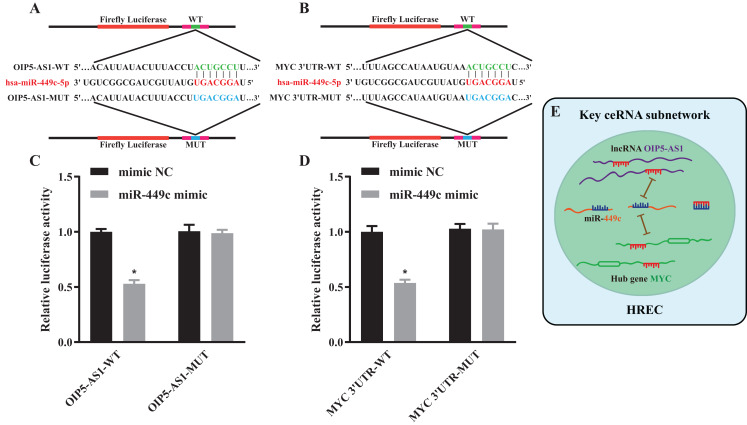
Luciferase reporter assay and a key ceRNA subnetwork. Schematic diagram illustrating the design of the luciferase reporters with WT or MUT for OIP5-AS1 (A) and MYC (B) The luciferase activity in HEK293T cells co-transfected with pmirGLO-OIP5-AS1-WT/MUT together with miR-449c mimic or mimic NC (C). The luciferase activity in HEK293T cells co-transfected with pmirGLO-MYC-WT/MUT together with miR-449c mimic or mimic NC (D). Hypothesis diagram of a key ceRNA subnetwork (E). *P < 0.05 versus the mimic NC + pmirGLO-OIP5-AS1/MYC-MUT groups.

We selected the top 5 DE lncRNAs and hub genes as candidate RNAs and verified their expression levels using the qRT-PCR method. The results suggested that ZNRD1-AS1, MEG3, OIP5-AS1, TPTEP1, MYC and SQSTM1 were significantly decreased and MAPK1 was significantly increased in the HG group (Figs. 6A and 6B*)*. Next, we discovered that the expressions of lncRNA OIP5-AS1 and the hub gene MYC were the most significantly down-regulated genes, and that miR-449c was the common target of both genes. We performed miR-449c expression verification and found that its expression was increased in the HG group (Fig. 6C). Finally, we performed a luciferase reporter assay to confirm that miR-449c was a common target of both genes and reconstructed a key ceRNA subnetwork (OIP5-AS1/miR-449c/MYC) (Fig. 7).

### OIP5-AS1 and MYC levels of hyperglycemic HRECs regulated by miR-449c in vitro

**Figure 8:**
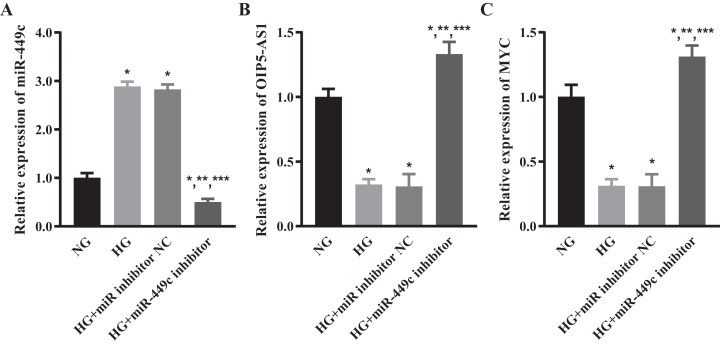
OIP5-AS1 and MYC levels of hyperglycemic HRECs regulated by miR-449c in vitro. qRT-PCR for miR-449c (A) OIP5-AS1 (B) and MYC (C) of HRECs in the NG, HG, HG transfection inhibitor NC and HG transfection miR-449c inhibitor groups. *P < 0.05 versus NG group; **P < 0.05 versus HG group; ***P < 0.05 versus HG transfection inhibitor NC group.

We tested the levels of miR-449c in the HG transfection miR-449c inhibitor group and HG transfection inhibitor NC group by qRT-PCR to ensure that the miR-449c inhibitor effectively reduced the expression of miR-449c in HRECs. The results showed that miR-449c in the HG transfection miR-449c inhibitor group was significantly lower than in the NG, HG and HG transfection inhibitor NC groups at 48 h, respectively. The levels of miR-449c in the HG group and HG transfection inhibitor NC group were higher than that in the NG group at 48 h (Fig. 8A). The expressions of OIP5-AS1 and MYC were measured by qRT-PCR to determine the levels of lncRNA OIP5-AS1 and hub gene MYC regulated by miR-449c in vitro. The results showed that OIP5-AS1 and MYC in the HG transfection miR-449c inhibitor group were significantly higher than in the NG, HG and HG transfection inhibitor NC groups at 48 h, respectively. The OIP5-AS1, MYC in the HG group, and HG transfection inhibitor NC group were lower than in the NG group at 48 h (Figs. 8B and 8C).

### Biological behaviors of HRECs modulated by miR-449c in vitro

**Figure 9:**
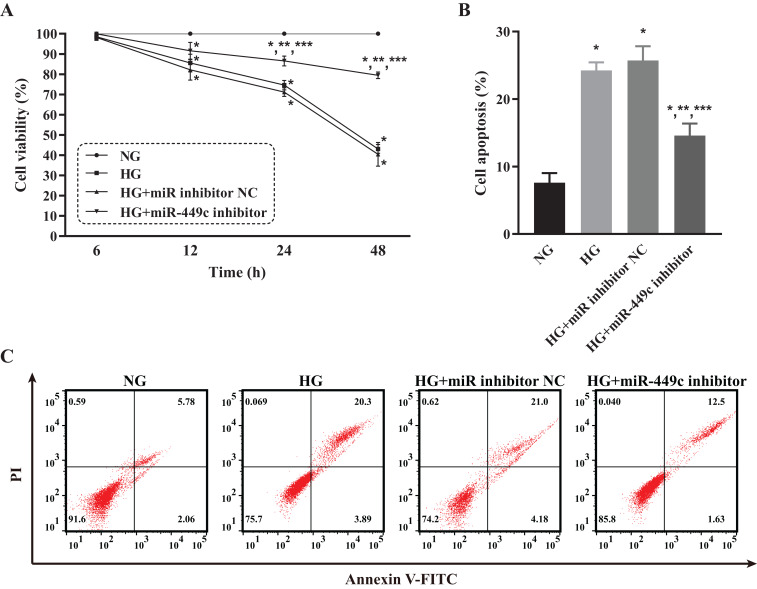
Biological behaviors of HRECs modulated by miR-449c in vitro. CCK-8 assay (A) and flow cytometry analysis (B and C) of HRECs in the NG, HG, HG transfection inhibitor NC and HG transfection miR-449c inhibitor groups. *P < 0.05 versus NG group; **P < 0.05 versus HG group; ***P < 0.05 versus HG transfection inhibitor NC group.

The viability and apoptosis of HRECs affected by miR-449c were analyzed by CCK-8 analysis and flow cytometry, respectively, to investigate the in-vitro biological behavior of miR-449c-regulated HRECs. The results showed that the cell viability of the HG group, HG transfection inhibitor NC group, and HG transfection miR-449c inhibitor group were all lower than that of the NG group at 12, 24 and 48 h. The cell viability of HRECs in the HG transfection miR-449c inhibitor group under hyperglycemic culture conditions was significantly greater than that of HRECs in the HG group and the HG transfection inhibitor NC group at 24 and 48 h. However, the cell viability of the three HG groups was not statistically significant at 6 and 12 h (Fig. 9A). Similarly, the cell apoptosis of the HG group, HG transfection inhibitor NC group and HG transfection miR-449c inhibitor group were all higher than that of the NG group at 48 h. Cell apoptosis of HRECs in the HG transfection miR-449c inhibitor group under hyperglycemic culture conditions was significantly lower than that of HRECs in the HG group and HG transfection inhibitor NC group at 48 h, respectively. However, cell apoptosis of HRECs in the HG group and HG transfection inhibitor NC group was not statistically significant at 48 h (Figs. 9B and 9C).

## Discussion

The development of DR is related to cell stress, hyperglycemia, and biochemical abnormalities. These factors cause multiple interactional networks of pathological and physiological changes that eventually result in pericyte necrosis and HREC dysfunction (Roy et al., 2017; Hammes, 2018). However, the molecular mechanism behind HG-induced HRECs damage remains unclear. Thanks to the rapid development of bioinformatics and high-throughput sequencing technology, we can discover the various abnormal expression of RNAs in HG HRECs.

Blood glucose levels critically impact the development of DR and various genes may be associated with DR (Petrovič, 2013). PPI network analysis is an important method used to determine gene function annotation. Multiple key genes were found in the PPI network constructed for this study, including MYC and MAPK1. MYC is a feature-rich gene linked to a variety of diseases. The expression of insulin-related genes and liver metabolism in DM can be regulated by MYC (Kaneto et al., 2002; Riu et al., 2002). MYC promotes the release of inflammatory cytokines in Müller cells in DR by adjusting the MIAT/TXNIP pathway (Zhang et al., 2019). MYC can reduce the expression of CDKN1A through active and passive recruitment of related inhibitory proteins, thereby affecting cell viability and apoptosis (Ball, 1997). According to our conclusion, MYC is involved in the G1/S transition of mitotic cell cycle, cell cycle G1/S phase transitionthe, and cell cycle pathway. After increasing the expression of MYC, the cell viability of the HG group increased than before. MAPK1 encodes a member of the MAP kinase family, also known as extracellular signal-regulated kinase 2 (ERK2). Its function involves multiple cell conduction pathways, which affects multiple cell functions, including proliferation, differentiation, and apoptosis (Keshet & Seger, 2010; Roskoski, 2012). Studies have shown that MAPK1 expression is increased in the retinal tissue of diabetic rats and it can be negatively regulated by MYC (Ye et al., 2010; Si et al., 2018). Therefore, these key genes affect each other and are associated with the pathogenesis of DR.

It is of clinical interest to determine how miRNAs regulate the occurrence and development of DR. Previous studies determined that the abnormal expression of miRNAs may be applied in a serological test as a potential biomarker in DR patients (Liu et al., 2018). We screened miR-449c as a hub node in the key subnetwork. The expression of miR-449c is generally reduced in cancer cells, including in gastric, liver and lung cancers. It also plays critical roles in adjusting the biological processes of cancer cells. Current evidence suggests that miR-449c can be widely involved in tumor cell growth, migration, proliferation and apoptosis (Miao et al., 2013; Sandbothe et al., 2017; Chen, Wang & Yue, 2018). We found that miR-449c was also upregulated in the HG group. By inhibiting its up-regulation, the cell viability increased and the apoptosis decreased under hyperglycemic conditions. Those lead us to believe that miR-449c may play a critical role in the development of DR by affecting HRECs.

Compounding evidence has suggested that the abnormal expression of lncRNAs may play an important role in the development of DR (Gong & Su, 2017). Therefore, it is essential to determine the molecular mechanism of lncRNAs to reduce the rate of vision loss of DR. ZNRD1-AS1, MEG3, OIP5-AS1 and TPTEP1 are the four key lncRNAs in the ceRNA network. ZNRD1-AS1 it is thought to be related to the progression of cancer (Li et al., 2016; Wang et al., 2017). Two recent studies showed that the expression of ZNRD1-AS1 is associated with the development and susceptibility of hepatocellular carcinoma and cervical cancer (Guo et al., 2015; Wen et al., 2015). However, there is no related research in DR and the expression of ZNRD1-AS1. We determined the expression of ZNRD1-AS1D in HRECs with HG and NG controls for the first time and its mechanism requires further exploration in DR. MEG3 is reduced in many cancer cell lines and tumor tissues (Zhang et al., 2010; Mondal et al., 2015; Peng et al., 2015; Zhuo et al., 2016). The overexpression of MEG3 has been found to promote tumor cell apoptosis and inhibit its in-vitro growth (Wang, Ren & Sun, 2012; Lu et al., 2013). The downregulation of MEG3 has been shown to enhance cell mobility, proliferation, and tube formation in endothelial cells (Liu et al., 2017). Research has shown that MEG3 can inhibit the NF-κB signaling pathway by targeting the miR-34a/SIRT1 axis, thereby reducing HG-induced inflammation and apoptosis. MEG3 can also prevent the development of DR by reducing the expression of TGF-β1 and VEGF (Zhang et al., 2018a; Tong et al., 2019). OIP5-AS1 is in the key ceRNA subnetwork and has a regulatory effect on certain diseases, affecting cell proliferation and apoptosis in cells such as hepatoblastoma and multiple myeloma cells. The knock-down of OIP5-AS1 can inhibit cell viability and increase apoptosis (Yang et al., 2017; Naemura et al., 2018; Zhang et al., 2018b). Its low expression in HRECs under hyperglycemic conditions was demonstrated for the first time in our study. TPTEP1 is also one of the DE lncRNAs. A previous study showed that it may be related to the invasion of hepatocellular carcinoma (Ding et al., 2019). However, there are no reports of its relationship with diabetes-related diseases and its role in DR needs further research. Therefore, the above key lncRNAs are novel in determining the pathogenesis of DR.

lncRNAs could function as ceRNAs to influence the inhibition effects of miRNAs on target genes (Qu et al., 2015). The study of ceRNAs may also provide targets for the development of novel drugs. We predicted and validated miR-449c’s role in reducing the expression of the target genes OIP5-AS1 and MYC. The expressions of OIP5-AS1 and MYC can be increased simultaneously by transfection with miR-449c inhibitors, thereby increasing the activity and reducing apoptosis of HRECs under hyperglycemic conditions. miR-449c may affect the expression of the target genes OIP5-AS1 and MYC, allowing OIP5-AS1 and MYC to perform their biological functions. The construction of the ceRNA network is unlike cell biology research or classical molecular research in that it focuses on specific molecular interactions designed to provide a more comprehensive understanding of the mutual regulatory mechanisms among RNAs that stem from HRECs under hyperglycemic culture conditions.

There were some limitations in this study. The sample size of the GSE122189 dataset was small, but we cannot change the original dataset, and the error of differential expression analysis using the limma software package would be amplified. Additional testing should be performed to verify our results. It is a conventional prediction method to predict the relationship of ceRNA through some definite databases. The predictive analysis based on the above database may also risk losing unknown genes. Finally, although our study preliminarily screened lncRNA OIP5-AS1 and miR-449c, further clinical in vivo and in vitro experiments are necessary to confirm its function mechanisms.

## Conclusions

DE lncRNAs and DE mRNAs were successfully filtered in the NG group and HG group, and ceRNA regulatory network and PPI network were constructed based on DE lncRNAs and DE mRNAs using the GSE122189 dataset of the GEO database for data analysis and mining. The genes in the PPI network were annotated by GO analysis and KEGG pathway enrichment analyses, and the expression of key RNAs was verified by qRT-PCR. The target relationship of OIP5-AS1/miR-449c/MYC was verified by the luciferase reporter assay and a key ceRNA subnetwork was constructed. The OIP5-AS1, miR-449c, and MYC were tested by transfecting miR-449c inhibitors, and the biological behavior of HRECs was discussed through cell viability and apoptosis. Our study led to a greater understanding of the effect of ncRNAs on HRECs under hyperglycemic conditions, and of the pathogenesis of DR.

## Supplemental Information

10.7717/peerj.9452/supp-1 Supplemental Information 1 QRT-PCR primers in this study.

10.7717/peerj.9452/supp-2 Supplemental Information 2 Differential profiling of lncRNAs and mRNAs in HG and NG-induced HRECs from microarray data.

10.7717/peerj.9452/supp-3 Supplemental Information 3 Construction of the ceRNA network.

10.7717/peerj.9452/supp-4 Supplemental Information 4 GO and KEGG pathway analysis of aberrantly expressed genes.

## Additional Information and Declarations

### Data Availability

The following information was supplied regarding data availability: The raw measurements are available in the Supplemental Files.
