# Supplementary material for: Integrative analysis of competitive endogenous RNA network reveals the regulatory role of non-coding RNAs in high-glucose-induced human retinal endothelial cells
Source: PeerJ. 2020 Jun 29;8:e9452. doi: 10.7717/peerj.9452 (PMC7331629; doi:10.7717/peerj.9452)
Supplement: Supplemental Information 1 [file peerj-08-9452-s001.docx]

Supplemental Table S1. qRT-PCR primers in this study.

| Gene Symbol |  | 5'-3' |
| --- | --- | --- |
| ZNRD1-AS1 | Forward | TCCTAGGATTGCTGCAGGTC |
|  | Reverse | CTATTGCCTGGATCCCATGT |
| MEG3 | Forward | CTGCCCATCTACACCTCACG |
|  | Reverse | CTCTCCGCCGTCTGCGCTAGGGGCT |
| OIP5-AS1 | Forward | TGCGAAGATGGCGGAGTAAG |
|  | Reverse | TAGTTCCTCTCCTCTGGCCG |
| TPTEP1 | Forward | CTGGGAGAAGTGCCCTTGC |
|  | Reverse | CACCTCATCAGTCATTTGCTCA |
| FER1L4 | Forward | CCGTGTTGAGGTGCTGTTC |
|  | Reverse | CCCATCCCAGGAGGTCACCT |
| MYC | Forward | TCTGGATCACCTTCTGCTGG |
|  | Reverse | TGTTGCTGATCTGTCTCAGG |
| MAPK1 | Forward | AGGCTGTTCCCAAATGCT |
|  | Reverse | CGTCACTCGGGTCGTAAT |
| CDKN1A | Forward | AAGTCAGTTCCTTGTGGAGCC |
|  | Reverse | GGTTCTGACGGACATCCCCA |
| MMP2 | Forward | GGAATGCCATCCCCGATAAC |
|  | Reverse | CAGCCTAGCCAGCCAGTCGGATTT |
| SQSTM1 | Forward | GCCTCTGGTTCTGACACTTT |
|  | Reverse | GGTGAGGTGGAAGGCATTTA |
| GAPDH | Forward | AGAAGGCTGGGGCTCATTTG |
|  | Reverse | AGGGGCCATCCACAGTCTTC |
| hsa-miR-449c-5p | Reverse Transcription | GTCGTATCCAGTGCAGGGTCCGAGGTGCACTGGATACGACACAGCCG |
|  | Forward | TGCGGUAGGCAGUGUAUUGCUAGCG |
|  | General primers | CCAGTGCAGGGTCCGAGGT |
| U6 | Reverse Transcription | GTCGTATCCAGTGCAGGGTCCGAGGTGCACTGGATACGACAAAATATGG |
|  | Forward | TGCGGGTGCTCGCTTCGGCAGC |
|  | General primers | CCAGTGCAGGGTCCGAGGT |
